# Urbanisation alters ecological interactions: Ant mutualists increase and specialist insect predators decrease on an urban gradient

**DOI:** 10.1038/s41598-020-62422-z

**Published:** 2020-04-14

**Authors:** Elise A. Rocha, Mark D. E. Fellowes

**Affiliations:** People and Wildlife Research Group, School of Biological Sciences, University of Reading, Whiteknights, Reading, Berkshire RG6 6AS UK

**Keywords:** Ecology, Urban ecology

## Abstract

The modification of habitats in urban areas is thought to alter patterns of species interactions, by filtering specialist species and those at higher trophic levels. However, empirical studies addressing these hypotheses remain limited in scope and number. This work investigates (1) how main urban land uses affect predator-prey and mutualistic interactions, and (2) how specialist and generalist predators respond to size and availability of urban green spaces. In a large town in the UK, experimental colonies of ant-attended Black bean aphid *Aphis fabae* and non-ant-attended Pea aphid *Acyrthosiphon pisum* were monitored over two years. Ants were more frequently found in highly urbanised sites; however mutualistic ants were also more often encountered when the habitat was more plant diverse. Aphids were not affected by urban land uses, but *A. fabae* numbers were positively related to the presence of mutualists, and so indirectly affected by urbanisation. Predators were the only group negatively affected by increased urbanisation, and specialist species were positively related to increased proportion of urban green areas within the habitats. While this work supports the hypothesis that specialist predators are negatively affected by urbanisation, we also show that a fundamental ecological interaction, mutualism, is affected by urbanisation.

## Introduction

Urbanisation is one of the defining environmental trends of recent times, almost completely modifying natural environments and significantly reducing local biodiversity^1,2^. Nevertheless, in most urban areas some native vegetation remains alongside introduced species within the urban matrix, mostly in parks and suburban gardens^3^, and their presence promotes biodiversity and provides ecosystem services (e.g. pollination, nutrient cycling). Urban areas can therefore be of value for biodiversity and conservation^4^. However, we have little understanding of how urbanisation modifies the patterns of species interactions.

At a simple level, urbanisation replaces natural systems with smaller sealed and impermeable areas, resulting in the reduction and fragmentation of habitat^5^. Beyond that, urbanisation changes key factors including local climate, nutrient availability, and disturbance levels^6^. Such factors change host plant quality, availability and accessibility (bottom-up factors), alter the abundance and diversity of natural enemies (top-down factors), and may modify the occurrence or intensity of mutualisms and competition (lateral factors)^7,8^. All such changes may alter how species interact, changing how ecological communities are structured in urban environments^9^.

Interactions between arthropod predators and their prey are particularly predisposed to being disrupted by urbanisation^8,10^, with specialist predators in particular likely to present a higher degree of sensitivity to the environmental disturbances that arises from habitat alteration^10–12^. It is not only consumptive interactions that may be affected by urbanisation. It is likely that mutualisms will also be affected^13^. The intensity and occurrence of mutualistic interactions can be strongly dependent on the physical and biological setting in which they occur^14,15^. Surprisingly, to our knowledge there are no studies that empirically evaluate how increased urbanisation might affect interactions between mutualists and other trophic groups (i.e. herbivores and enemies). Populations of insect herbivores, such as aphids and associated natural enemies and mutualists, can be used as a model system to address such questions.

Aphids are widespread and abundant in urban habitats^16^. Their populations are structured by host plant quality and availability, natural enemies, and for some species, interactions with ant mutualists^17^. Bottom-up effects are the consequence of variation in host plant diversity, quality and structure e.g.^18^. Top down effects are the result of the action of both generalist and specialist natural enemies, such as spiders, parasitoids, coccinellid beetles and hoverflies e.g.^19^. Lateral factors include the presence of mutualists; some aphid species are mymercophiles, tended and protected by ants in return for honeydew^20^. Each group of interactions may be affected by the biotic and abiotic changes typical of urban ecosystems, allowing us to tease apart how urbanisation may affect predator-prey and mutualistic interactions.

In this study, we are using a tri-trophic system of aphids and their associated predators and mutualistic ants to ask how the main land uses that compose cities (gardens, woodlands, roads and buildings), and the plant species richness of urban green areas affects aphid population numbers and the presence (or absence) of the mutualistic ants and predators associated with aphid colonies. Our study system was composed of two host plants (*Vicia faba* L.), one carrying a colony of *Acyrthosiphon pisum* Harris and the other a colony of *Aphis fabae* Scopoli, which were placed on a gradient of urbanisation in a large town in southern England. Both aphid species are known to suffer heavy predation^20,21^, but in contrast to *A. pisum*, *A. fabae* is regularly ant-attended. El‐Ziady and Kennedy^22^ demonstrated that the ant *Lasius niger* Linnaeus attending *A. fabae* accelerated the rate of multiplication and growth of the aphid colony and decreased the proportion of winged (dispersing) individuals among the adults. These ants showed “ownership behaviour”, acting aggressively against intruders such as ladybirds and hoverfly larvae. However, Pontin^23^ demonstrated that *Lasius* species regularly prey on aphids of non-myrmecophilus species. As such, our second question is how urban habitat features and plant richness can mediate interactions between aphids, predators and ants, given the behaviour of ants defending *A. fabae* against its predators, and also the potential competitive interspecific interactions of ants that prey on *A. pisum* with other predatory species. Lastly, we ask if specialist and generalist predators respond differently to the amount of green spaces present in the urban area.

## Results

In total, we observed 18490 *Acyrthosiphon pisum* Harris (the pea aphid, hereafter PA) aphids and 46804 *Aphis fabae* Scopoli (the black bean aphid, hereafter BB) aphids, 377 PA predators and 374 BB predators, 244 ants preying on PA colonies and 1555 mutualistic ants on BB colonies.  Two ant species, *Myrmica rubra* (L.) and *Lasius niger* (L.), were found on PA and BB colonies. Parasitized aphids were found in negligible numbers (not found on PA colonies and only found on period 3, 4 and 7 on BB colonies in few study sites). Analysis of the latter is reported in Rocha and Fellowes^24^. The proportion of habitat elements and their maximum and minimum values are shown in Table 1.

**Table 1:** Mean proportion (±SE) and range values of habitat elements within 30 meters buffers of the study sites.

	Plant richness	Roads	Buildings	Woodland	Gardens	Green areas
Mean (±SE)	34.90 ± 1.24	0.287 ± 0.014	0.122 ± 0.008	0.191 ± 0.024	0.349 ± 0.016	0.545 ± 0.018
Range	14–100	0–0.774	0–0.463	0–1	0–0.719	0–0.848

### Urban land use and interactions between aphids, ants and predators

Numbers of predators were positively correlated with aphid numbers (Table 2, models 3 and 4). The presence of predatory ants did not affect PA numbers, and the presence of mutualistic ants was associated with increased numbers of BB (model 2). Habitat features did not significantly affect aphid numbers (models 1 and 2). The presence of ants negatively affected the likelihood of finding predators on colonies of both aphid species (models 3 and 4). Fewer BB predators were found in areas with a higher proportion of roads, and an increased proportion of buildings in the habitat negatively affected the presence of PA predators, but positively affected the numbers of predatory ants, found on PA colonies (models 3, 4 and 5). Increased numbers of mutualistic ants on BB colonies were associated with increased BB colony size, higher local plant species richness and higher proportions of roads in the study sites (model 6).

**Table 2:** Summary of models predicting abundance of *Aphis fabae* (BB) and *Acyrthosiphon pisum* (PA) and the occurrence of predators and ants found on colonies of each aphid species as response variables, and proportion of habitat types, plant richness and aphid species, predators and ants as explanatory variables.

Model ID	AIC	Response variable	Explanatory variable	Coefficient value ± SE	P
1	304.6	**PA aphid**	Intercept	1.103 ± 0.196	0.0000
			**Presence of predators**	0.556 ± 0.090	0.0000
			Proportion of buildings	0.549 ± 0.346	0.1152
2	279.4	**BB aphid**	Intercept	1.862 ± 0.185	0.0000
			**Presence of predators**	0.188 ± 0.078	0.0165
			**Presence of ants**	0.440 ± 0.078	0.0000
			Plant richness	−0.003 ± 0.002	0.1151
			Proportion of buildings	0.557 ± 0.316	0.0796
3	182.9	**PA predators**	Intercept	−1.101 ± 0.551	0.0457
			**PA aphid**	1.664 ± 0.551	0.0000
			**Presence of ants**	−1.341 ± 0.502	0.0076
			Proportion of buildings	−3.320 ± 1.743	0.0568
4	212.2	**BB predators**	Intercept	0.342 ± 0.638	0.5920
			**BB aphid**	0.790 ± 0.316	0.0124
			**Presence of ants**	−1.119 ± 0.407	0.0060
			**Proportion of roads**	−2.120 ± 0.926	0.0221
5	163.3	**PA ants**	Intercept	−1.545 ± 0.420	0.0002
			**Presence of predators**	−1.147 ± 0.450	0.0108
			**Proportion of buildings**	5.169 ± 1.682	0.0021
6	185.9	**BB ants**	Intercept	−6.627 ± 1.229	0.0000
			**BB aphid**	2.087 ± 0.419	0.0000
			**Presence of predators**	−0.888 ± 0.408	0.0294
			**Plant richness**	0.036 ± 0.011	0.0015
			**Proportion of roads**	2.042 ± 1.013	0.0438

AIC values for each model are given. Models with significant explanatory factors are shown in bold.

### Relationship between generalist/specialist predators and urban green space

**Figure 1:**
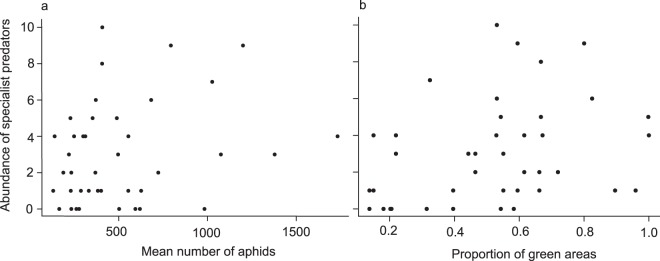
Abundance of specialist predators found on *Aphis fabae* and *Acyrthosiphon pisum* colonies according to (**a**) the abundance of aphids and (**b**) the proportion of green space in study sites.

Generalist predators were more common than specialists (W = 538.5, Z = 2.38, P < 0.05; median values of generalists and specialists recorded per colony were 2 and 4 respectively). The best model in explaining the abundance of specialist predators had two positive and significant explanatory factors, the mean number of aphids and the proportion of green areas on the study sites (Table 3, model 1; Fig. 1). The best model in explaining the abundance of generalist predators had only one variable, proportion of green areas on the study sites; however, this factor was not statistically significant (Table 3, model 2).

**Table 3:** Summary of models predicting the abundance of specialist predators (model 1) and generalist predators (model 2) found on both *Aphis fabae* and *Acyrthosiphon pisum* colonies.

Model ID	AIC	Response variable	Explanatory variable	Coefficient value ± SE	P
1	199.3	Specialist predators	Intercept	−2.138 ± 0.915	0.019
			**Mean number of aphids**	0.969 ± 0.319	0.002
			**Proportion of green areas**	1.189 ± 0.391	0.002
2	41.8	Generalist predators	Intercept	0.499 ± 0.130	0.001
			Proportion of green areas	0.383 ± 0.224	0.096

AIC values for each model are given. Models with significant explanatory factors are shown in bold.

## Discussion

In this study we asked how habitat changes associated with urbanisation may alter patterns of interactions between insect herbivores, their predators and mutualistic ants. Additionally, we wanted to investigate if the amount of green space in urban environments is an important environmental factor selecting species of specialist or generalist aphid predators. We controlled for habitat associated variation in plant quality and initial aphid colony size, allowing us to disentangle effects resulting from changes in the presence of natural enemies and mutualist ant species. Local habitat factors (human-constructed surfaces, local plant species richness) did not affect aphid colony size, but the presence of ant mutualists was associated with an increase in numbers of black bean aphids. The presence of ants was associated with a reduction in insect predators, and both were more likely to be found on larger aphid colonies. Predator occurrence was reduced at study sites with higher proportions of roads (BB) and buildings (PA). However, ants showed the inverse pattern, where increased numbers of predatory ants (PA) were associated with an increased proportion of buildings, and attendance of BB colonies by mutualistic ants was positively associated with the proportion of roads and plant species richness. This suggests that anthropogenic changes associated with urbanisation may alter the structure of local ecological assemblages, with some taxa (predatory and mutualistic ants) benefiting more than others (specialist insect predators). A difference between numbers of specialist and generalist predator groups was also observed, as higher numbers of specialist predators are correlated to greater proportion of green areas in urban habitats, while generalist predators did not follow this trend. These findings are consistent with studies showing high sensitivity of monophagous and oligophagous species to urbanisation, habitat fragmentation and habitat loss^11,25–29^, but this is the first study to show that urbanisation changes the likelihood of such a mutualistic interaction.

We found that predators were reduced in areas with increased proportions of roads. Fundamentally, roads can act as barriers or filters to animal dispersal^30^, with studies finding that carabid beetles and wolf spiders are blocked by roads as narrow as 2.5 m wide^31^. Furthermore, the quantity and extent of impervious cover (paved surfaces structures such as buildings and roads) cause strong detrimental effects to arthropod diversity and abundance, including natural enemies such as parasitoids^32–34^. Specialist predators are linked to the presence of resources utilised by their prey, consequently, loss of prey habitat would also mean reduction of predator habitat^35,36^. Here we found that numbers of specialist predators were positively linked to the amount of local green space, but that numbers of generalists were not similarly affected. Overall, environmental changes are expected to be more disadvantageous to specialist species, as generalists are better able to adapt to varying habitat conditions and prey availability^29,37,38^.

Predatory ants found on PA colonies and mutualistic ants found on BB colonies were positively associated with the proportion of roads and buildings in the habitat, respectively. The presence of mutualistic ant-aphid interactions was positively associated with more plant diverse sites; aphid diversity in urban gardens is associated with plant diversity and abundance^16^. Urbanised areas may serve as habitat and corridors for dry-adapted and heat tolerant species such as ants^39,40^, and such habitats select for opportunistic, highly competitive ant species^41,42^. *Lasius niger* and *Myrmica rubra* live in colonies of several thousand individuals, showing aggressiveness and displacement against competitors^43,44^, both species are omnivores with varied diet which consists of honeydew, other invertebrates, pollen, seeds and human waste^45^ and are good candidates for benefitting from urban habitats^46^. Indirect interactions between ants and other natural enemies can be complex^47^. However, the negative effect found where both predatory and mutualistic ants displaced other predator species on our experimental aphid colonies was not surprising. In our study sites ants acted as predators on PA colonies, displaced predators of both aphid species, and acted as beneficial mutualists of BB aphids. This behaviour could be linked to the fact that ants not exclusively choose to just tend aphids or just prey on them, but as whether ants tend aphids for honeydew or eat them and their rate of attendance, depends upon food availability in the ants’ foraging areas^20,48,49^. According to Pontin^23^ ants would keep a balanced protein-carbohydrate food intake by initiating predation on attended aphids when other prey were in short supply, and though the study of Offenberg^48^ did not support Pontin’s hypothesis - who observed that when offered alternative sugar, the interaction moved from mutualism to exploitation due to decreased ant-tending and increased predation, and alternative prey had no significant effect – their work was not performed on “real life” habitats but in controlled laboratory conditions where only three different alternative prey were offered. The fact that ants with predatory behaviour against PA aphids were more likely to be found in highly urbanised habitats with increased proportions of buildings may also indicate an increased need for prey and protein rich food sources by ants in highly urbanised environments.

Little consideration has been given to the effects of habitat structure of cities in determining trophic dynamics and species interactions. Our data suggests that such variables play a major role for predatory and mutualistic interactions, with likely consequences for the structuring of urban insect communities. The reduction in specialist predator numbers, with a concurrent increase in ant presence, could also lead to a potential increase in herbivore populations of ant-attended species. This may affect the environmental services predators provide^50,51^. In our work we found that numbers of specialist predators were positively linked to the amount of local green space, but that numbers of generalists were not affected by the same variable. Some studies have found a constant number of generalist predators on gradients of human disturbance^11,52^, however others have found that generalists are even more abundant in cities than specialists^28,53^. There is a strong theoretical expectation that generalist and specialist predators will have distinctive responses associated with changes in habitat^37^. Overall, environmental changes are expected to be more disadvantageous to specialist species, as generalists are able to adapt more easily to varying habitat conditions^29,38^. Thus specialist aphid predators may particularly benefit from the increased amount of potential habitat promoted by the presence of urban green spaces.

Urbanisation is transforming the areas where most of the world’s people live. Understanding how patterns of species interactions change in such radically altered environments is of critical importance if we are to develop approaches to help maintain biodiversity in highly altered, novel environments. Here we show that for two abundant and widespread species of aphids, their specialist predators are lost from assemblages as urbanisation increases, but no such pattern is seen with generalists. We also observed that a facultative mutualism (ant attendance) increased with urbanisation, suggesting that urban areas might act as filter that benefits dominant ant species that thrive when tending aphid colonies. Together, these results suggest that insect communities in urban areas are not just simply depauperate, but how they are structured may differ. Urban ecosystems are indeed novel ecosystems, but just how novel they are remains to be fully explored.

## Methods

### Study area and habitat variables

**Figure 2:**
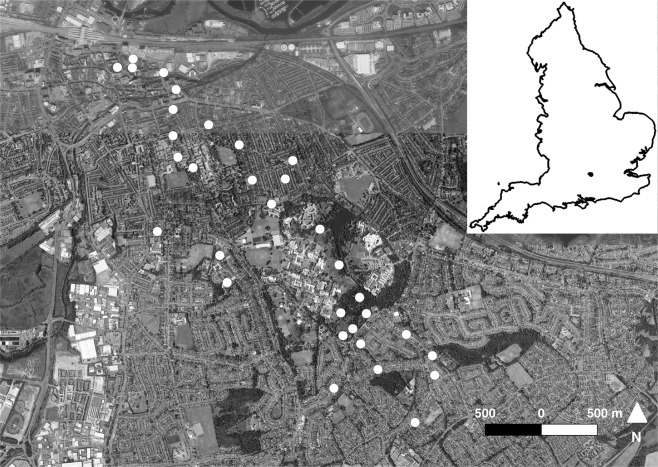
Study site location in Greater Reading, England (*n* = 32). Aerial image was obtained from Digimap EDINA Aerial. Figure created using QGIS 2.8.1^55^.

The study area is located in Greater Reading, Berkshire, UK (51°27′N, 0°58′W, Fig. 2). Reading is a large town with a population of 290 000, which covers an area of ca. 72 km^2^^54^. For the first year of sampling, 27 experimental sites were studied and 32 sites in the second year. Site selection captured a gradient from highly urbanised sites on the town centre to suburban areas closer to rural areas located on the south^24^. Each study site was at least 110 meters apart. Habitat variables were obtained using GIS, utilising the topography layer from Digimap EDINA MasterMap, at a scale of 1:1250. Thirty meters radius buffers were delimited in each study site, and a reclassification of vectors was made to calculate proportions of area of the following habitat types within those buffers: green areas (gardens and parks), woodlands (shrubs and trees), and impervious surfaces, made up of buildings (any artificial structure made of concrete, brick or stone) and roads (roads, roadsides, tracks or paths made of surfaces such as asphalt), using QGIS 2.8.1^55^. Additionally, plant species richness within the 30 meters radius buffers of each study site was estimated by counting plant morphospecies (defined as taxonomic groups which could be separated by eye in the field by a trained botanist). Thirty meters radius buffers were chosen as the optimal size to account for significant local habitat variation between study sites while solving practicalities regarding site access for plant diversity estimation.

### Study systems and summer recording

Monoclonal cultures of *Acyrthosiphon pisum* Harris (PA) and *Aphis fabae* Scopoli (BB) were maintained in a laboratory using plastic and mesh cages. All cultures and experimental colonies were identically reared in a Controlled Temperature room at 20 ± 1 °C, 16:8 L:D h light regime and at ambient humidity on broad bean, *Vicia faba* L. (var. the Sutton dwarf). Plants were sown in pots with potting compost (Vitax Grower, Leicester, England) and watered as needed.

Three days before being allocated to the study sites, three adults from each aphid species were transferred from the monoclonal cultures and reared in cages containing 14 to 16-day-old broad bean plants (18–22 cm tall), to allow the colonies to become established. After three days, one colony of PA and one of BB on *Vicia faba* plants were placed at each study site (60–80 cm apart). Two days after the experimental colonies were placed in the field, aphid, ant and predator numbers were recorded for the first time, and recording subsequently occurred every four days, for five recording events in total. At the end of this sampling period, colonies were replaced. Sampling was repeated four times in 2015 (period one: May 16, 20, 24, 28 and June 1; period two: June 15, 19, 23, 27 and July 1; period 3: 16, 20, 24, 28 July and August 1; period four: August 14, 18, 22, 26 and 30), and three times in 2016 (period five: May 16, 20, 24, 28 and June 1; period six: June 16, 20, 24, 28 and July 2; period seven: July 29, and August 2, 6,10 and 14).

### Data analysis

All statistical analyses were carried out using R 3.1.2^56^.

#### Urban land use and interactions between aphids, ants and predators

Here, the dataset consisted of the cumulative numbers of aphids and the presence or absence of predators and ants in the five counting events on each of the seven sampling periods. Some colonies were lost during the experiment (due to poor plant health, herbivory, damage or theft by the public). This resulted in 183 observations of BB colonies and 177 observations for PA colonies. To analyse PA and BB aphid colony numbers we applied separate linear mixed models fitted by reduced maximum likelihood using package nlme^57^, and as explanatory variables we used presence or absence of ants and predators, proportion of gardens, buildings, roads, and plant species richness. Counts of aphids were log_10_-transformed to deal with extreme values and to standardize and homogenize model residuals. For these models we accounted for repeated sampling of the colonies through time by adding period as a random effect. We removed the proportion of woodland from the set of explanatory variables due to its correlation with garden and roads (−0.66 and −0.61, respectively).

To investigate which biotic and abiotic factors determined the occurrence of predators and ants on PA and BB colonies we performed separate logistic regression mixed models with a binomial error distribution (with canonical link logit) using the function glmer of package lme4^58^, fitted by maximum likelihood^59^. When modelling predators we used as explanatory factors the proportion of gardens, buildings, roads and plant species richness, number of aphids and presence or absence of ants. When modelling ants we used the same habitat variables, as well as controlling for aphid numbers on the colonies and the presence or absence of predators.

#### Relationship between generalist/specialist predators and urban green space

Predators found attacking aphid species were summed together and classified according to Rotheray^20^ into specialists (obligate aphid predators) and generalists (opportunistic aphid predators). Ladybirds (Coccinellidae), lacewings (Chrysopidae), flower bugs (Anthocoridae), aphid midges (Cecidomyiidae) and hoverfly (Syrphidae) larvae were considered as specialist aphid predators; earwigs (Dermaptera), ground beetles (Carabidae), spiders (Araneae) and harvestmen (Opiliones) were considered as generalist aphid predators. In order to obtain a meaningful quantitative response and avoid an excess of zeroes, the dataset of the two sampling periods with higher predator numbers in 2015 (period one and two) and the other two periods with highest predator numbers of 2016 (sampling periods five and seven) were summed together. As some colonies of both aphid species were damaged across different sampling periods, they were discarded from the dataset, leaving 41 observations.

To address possible differences in abundance between numbers of specialist and generalist predators, we used a paired Wilcoxon signed-rank test^59^. In order to assess the effect of urban green spaces on numbers of specialist predators a generalised linear mixed method (GLMM) fitted by maximum likelihood (Laplace Approximation), with a Poisson error distribution and a log link function was performed^60^, relating numbers of specialist predators to the proportion of green spaces (i.e. sum of the proportions of gardens and woodlands within 30 m buffers), occurrence of ants and mean number of aphids, using package lme4^58^. As numbers of generalist predators were over-dispersed, this variable was log_10_ transformed and then related to the proportion of green spaces, occurrence of ants and mean number of aphids using a linear mixed effect model fitted by reduced maximum likelihood on package nlme^57^. For these models, year of sampling was considered as a random factor, and mean numbers of aphids were log_10_ transformed in order to deal with extreme values and improve model convergence^60^.

For all analyses, model selection was done by model comparison using Akaike’s Information Criteria (AIC) by fitting the full model with the set of all explanatory variables and removing the least significant term on each step (refitting the model each time), until the optimal model is found^59,60^. We checked for collinearity between explanatory variables in all models through variance inflation factors (VIF), with VIF values higher than 3 indicating that covariation between predictors may impose a problem^60^. Our VIF values were in the range of 1.01–1.50. The response variables and model residuals were checked for spatial autocorrelation through spline correlograms on package ncf^61^, in which we did not find any significant spatial structure. We also confirmed the validity of models by checking normality, independence and homogeneity of model residuals.
